# Estimating Sampling Selection Bias in Human Genetics: A Phenomenological Approach

**DOI:** 10.1371/journal.pone.0140146

**Published:** 2015-10-09

**Authors:** Davide Risso, Luca Taglioli, Sergio De Iasio, Paola Gueresi, Guido Alfani, Sergio Nelli, Paolo Rossi, Giorgio Paoli, Sergio Tofanelli

**Affiliations:** 1 National Institute on Deafness and Other Communication Disorders, NIH, Bethesda, MD 20854, United States of America; 2 Laboratory of Molecular Anthropology and Centre for Genome Biology, Department of BiGeA, University of Bologna, via Selmi 3, 40126 Bologna, Italy; 3 Dipartimento di Biologia, University of Pisa, Via Ghini 13, 56126 Pisa, Italy; 4 Dipartimento di Genetica Biologia dei Microrganismi Antropologia Evoluzione, University of Parma, Parco Area delle Scienze 11/a, 43124 Parma, Italy; 5 Dipartimento di Scienze Statistiche, University of Bologna, Via Belle Arti 41, 40126 Bologna, Italy; 6 Bocconi University, Dondena Centre and IGIER, Milan, Italy; 7 Archivio di Stato, Lucca, Italy; 8 Dipartimento di Fisica, University of Pisa, Largo Bruno Pontecorvo 3, 56127 Pisa, Italy; Estonian Biocentre, ESTONIA

## Abstract

This research is the first empirical attempt to calculate the various components of the hidden bias associated with the sampling strategies routinely-used in human genetics, with special reference to surname-based strategies. We reconstructed surname distributions of 26 Italian communities with different demographic features across the last six centuries (years 1447–2001). The degree of overlapping between "reference founding core" distributions and the distributions obtained from sampling the present day communities by probabilistic and selective methods was quantified under different conditions and models. When taking into account only one individual per surname (low kinship model), the average discrepancy was 59.5%, with a peak of 84% by random sampling. When multiple individuals per surname were considered (high kinship model), the discrepancy decreased by 8–30% at the cost of a larger variance. Criteria aimed at maximizing locally-spread patrilineages and long-term residency appeared to be affected by recent gene flows much more than expected. Selection of the more frequent family names following low kinship criteria proved to be a suitable approach only for historically stable communities. In any other case true random sampling, despite its high variance, did not return more biased estimates than other selective methods. Our results indicate that the sampling of individuals bearing historically documented surnames (founders' method) should be applied, especially when studying the male-specific genome, to prevent an over-stratification of ancient and recent genetic components that heavily biases inferences and statistics.

## Introduction

The question of how representative is a sample of a given population is particularly critical in human genetic research, where data collection is strongly affected by ethical and social factors. The choice of the sampling strategy can affect the estimation of a number of relevant parameters and, accordingly, the inferences one can make about either the groups or the genetic variants under study. The ideal goal is to achieve the best compromise among recruitment costs, statistical power and representativeness. To make a sample an unbiased proxy of the underlying population, either for genetic screening, genotype-phenotype association studies or evolutionary reconstructions, its composition should minimize close kinship, ambiguous individual ancestry assignments, and the side effects of population stratification.

Most of present-day human populations have experienced recent admixture to some extent [1] and genetic flows make it difficult to discern ancestral genotypes and alleles, stabilized by many generations of historical residency and reinforced by endogamy, from genotypes and alleles introduced by recent migration. In population-based association studies, methodological corrections have been widely used to overcome spurious associations driven by undetected genetic structure. All of these methods have restrictions and drawbacks [2, 3]. In human evolutionary genetics, especially when inferring past demography upon Y chromosome diversity, direct methods based on either self-declared ancestry or a number of selective criteria based on surnames are routinely adopted, but the reliability of these methods has not been assessed so far. Owing to the co-segregation of surnames and Y markers in the Occidental practice, the conclusions inferred from samplings based on family names should be intended as specifically referred to the male-specific genome with the corollary that the higher the parents’ co-ancestry (marital kinship) of a community, the more they could be extended to whole genome studies. The use of donors with surnames documented in the early historical records of a given community allows obtaining genetic profiles not affected by recent shuffling. In addition, the adhesion to the oldest surname distribution of a community can provide the null hypothesis, in order to test how fully a sample reflects the ancestral genetic composition. Unfortunately, this method is rarely adopted due to difficulties in retrieving historical records. The so-called “grandparents”, or “two-generation residency”, criterion gives a picture of the state of a population about a century earlier than the sampling time and generally depends on the accuracy of self-declarations. Selecting only surnames whose distribution is limited to a relatively small area of origin (geographically restricted surnames), with or without the aid of neural network algorithms [4, 5], helps discarding potentially polyphyletic lineages. The selection of donors with the most frequent surnames, assuming that the most common surnames represent lineages of long residency, and the random drawing from the current distributions of family names, are two other widely used methods [6, 7, 8].

Using surnames to select research subjects has the advantage that family names are cultural markers typically transmitted from fathers to sons in most human societies. The distributions and mode of inheritance of surnames mirror the non-recombinant markers of the male specific regions of the Y chromosome (MSY). The main difference between surnames and MSY sequences is the nature of the transmitted units which are predominantly, if not exclusively, identical by descent for MSY markers and identical by state (polyphyly) other than by descent (monophyly) for surnames. In addition, the depth of the genealogical reconstruction is largely unbounded for MSY markers, while for surnames it is limited to the time since such family names have been stabilized as markers of inheritance (around 1200–1800 AD in Western societies). Such differences have been invoked to explain deviations from neutral expectations when surnames were used to estimate consanguinity or gene propagation [9, 10]. Nonetheless, the long-term dynamics of both natural and social phenomena tend to scale frequency spectra and are subject to the same laws of parameterization [11]. With few exceptions [12], within the last 20–30 generations surnames and MSY markers can be confidently considered as neutral alleles evolving under the same stochastic model with negligible effects from mutation and selection. Hence, last names can be considered as cost-efficient estimators of recent demographic changes occurred by migration and size fluctuations.

A number of reasons makes the Italian population an ideal case-study for surnames-related research: Italians show one of the highest surname diversity in the world [8] with a mean number of bearers per surname approaching twenty. In addition, historical documents are available since the Middle Ages for many communities, and standardized religious population registers, with barely the same geographic relevancy than present-day Italy, have been maintained since the Council of Trent (1545–1563 AD). Lastly, genetically and culturally closed communities still survive in proximity to open populations [13].

The present work aims at first comparing different surname-based sampling strategies, to estimate the bias associated with each method. In order to do this, we reconstructed the surname distributions of 26 Italian communities with different demography in the time interval encompassing the 1447–2001 AD. The degree of overlapping between “reference core” surname distributions, obtained from analyzing the oldest historical frames, and the distributions obtained from sampling present-day surnames by probabilistic (random) and selective (based on historically resident, locally spread and more frequent surnames) methods was quantified at different geographic scales (municipality, region, Country). Whenever possible, jackknife re-sampling and comparisons between different data sources and models, with low and high levels of kinship, were applied to weigh the various sources of the sampling bias.

## Materials and Methods

### Populations and records

**Fig 1:**
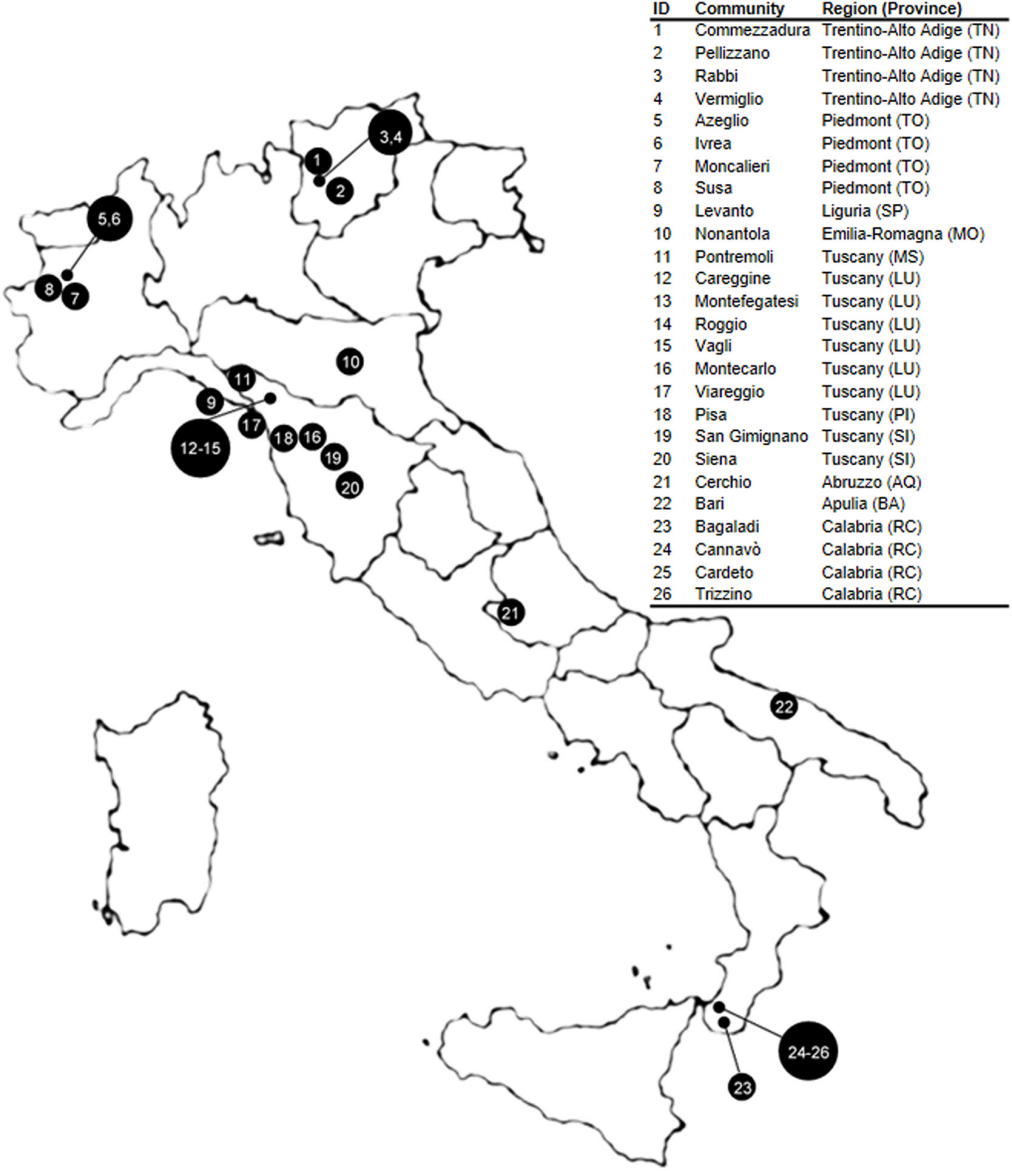
Map of Italy showing the location of the 26 investigated communities. This Fig is similar, although not identical, to the original image, and is therefore for illustrative purposes only.

Twenty-six Italian communities belonging to 11 provinces and 8 regions were analyzed (Fig 1): four of them are located in Trentino-Alto Adige [Rabbi (TN), Pellizzano (TN), Commezzadura (TN), Vermiglio (TN)], four in Piedmont [Ivrea (TO), Azeglio (TO), Moncalieri (TO), Susa (TO)], one in Liguria [Levanto (SP)], one in Emilia-Romagna [Nonantola (MO)], ten in Tuscany [Viareggio (LU), Pisa (PI), Siena (SI), San Gimignano (SI), Roggio (LU), Careggine (LU), Vagli (LU), Montefegatesi (LU), Pontremoli (MS), Montecarlo (LU)], one in Abruzzo [Cerchio (AQ)], one in Apulia [Bari (BA)] and four in Calabria [Bagaladi (RC), Cannavò (RC), Cardeto (RC), Trizzino (RC)]. Table 1 recapitulates the relevant data for each of the investigated communities, which are widely dispersed over the peninsula at different altitudes, differing in size, growth rate, and time since the oldest repertoire of surnames.

**Table 1:** Details of the studied communities/municipalities. N_0_, number of individuals of the oldest list of surnames; N_t_, number of individuals of the present-day list of surnames; altitude is indicated in meters above sea level; relative growth rate calculated as N_t_-N_0_/N_t_.

Community	Region (Province)	Oldest Historical Source	Recent Source	N_o_	N_t_	S_o_	S_t_	Altitude	Growth rate
Commezzadura	Trentino-Alto Adige (TN)	Marriage acts 1700	SEAT 1993	297	350	44	166	850	0.15
Pellizzano	Trentino-Alto Adige (TN)	Marriage acts 1700	SEAT 1993	548	324	85	134	925	-0.69
Rabbi	Trentino-Alto Adige (TN)	Marriage acts 1566	SEAT 1993	292	482	88	103	1,095	0.39
Vermiglio	Trentino-Alto Adige (TN)	Marriage acts 1714	SEAT 1993	341	462	28	68	1,261	0.26
Azeglio	Piedmont (TO)	Baptismal acts 1543	SEAT 1993	895	423	100	217	260	-1.12
Ivrea	Piedmont (TO)	Census Paper 1613	SEAT 1993	3,835	9,816	568	4,861	253	0.61
Moncalieri	Piedmont (TO)	Census Paper 1613	SEAT 1993	6,129	20,436	776	9,382	219	0.7
Susa	Piedmont (TO)	Census Paper 1613	SEAT 1993	4,447	2,283	341	1,276	503	-0.95
Levanto	Liguria (SP)	Census Paper 1662	SEAT 1993	1,728	5,716	329	1,151	3	0.7
Nonantola	Emilia-Romagna (MO)	Census Paper 1629	SEAT 1993	3,451	3,407	181	1,092	24	-0.01
Careggine	Tuscany (LU)	Marriage acts 1566	SEAT 1993	243	206	115	70	882	-0.18
Montecarlo	Tuscany (LU)	Baptismal acts 1527	SEAT 1993	3,913	1,226	283	545	162	-2.09
Montefegatesi	Tuscany (LU)	Marriage acts 1600	ISTAT 1991	398	270	108	55	842	-0.47
Pisa	Tuscany (LU)	Baptismal acts 1447	SEAT 1993	17,504	35,921	1,830	10,913	4	0.51
Roggio	Tuscany (LU)	Marriage acts 1775	ISTAT 1991	115	175	30	41	858	0.34
San Gimignano	Tuscany (LU)	Marriage acts 1700	SEAT 1993	290	2,357	73	1,013	324	0.88
Siena	Tuscany (LU)	Census Paper 1767	SEAT 1993	2,941	56,956	1,373	5,626	322	0.95
Vagli	Tuscany (LU)	Marriage acts 1700	SEAT 1993	553	379	96	100	575	-0.46
Viareggio	Tuscany (LU)	Census Paper 1705	SEAT 1993	290	57,514	86	7,263	2	0.99
Pontremoli	Tuscany (MS)	Marriage acts 1559	SEAT 1993	249	3,400	61	976	236	0.93
Cerchio	Abruzzo (AQ)	Census Paper 1700	SEAT 1993	932	1,735	144	146	834	0.46
Bari	Puglia (BA)	Census Paper 1598	SEAT 1993	8,872	111,221	1,065	12,993	5	0.92
Bagaladi	Calabria (RC)	Baptismal acts 1657	SEAT 1993	125	399	31	120	460	0.69
Cannavò	Calabria (RC)	Baptismal acts 1601	ISTAT 2001	994	3,935	274	577	147	0.75
Cardeto	Calabria (RC)	Baptismal acts 1670	SEAT 1993	924	695	129	129	700	-0.33
Trizzino	Calabria (RC)	Baptismal acts 1706	ISTAT 2001	137	104	34	28	551	-0.32

TN, Trento; TO, Turin; SP, La Spezia; MO, Modena; LU, Lucca; MS, Massa and Carrara; AQ, L’Aquila; BA, Bari; RC, Reggio Calabria.

We reconstructed the earlier distributions of surnames from either three kinds of historical records: census (*Stato delle anime*), baptismal (*Atti battesimali*), and marriage (*Atti matrimoniali*) sacramental registers. The present-day distributions of surnames from the same communities were extracted from the digitalized versions of the 1993 complete national phone directory [14] and/or from the 1991 and 2001 National Institute of Statistics (ISTAT) [15] records. All the original historical sources of data are public and can be accessed upon request from the relevant institutional archives: diocesan and /or civil anagraphical archives of Azeglio, Bagaladi, Bari, Cannavo’, Cardeto, Careggine, Cerchio, Commezzadura, Ivrea, Levanto, Moncalieri, Montecarlo, Montefegatesi, Nonantola, Pellizzano, Pisa, Pontremoli, Rabbi, Roggio, San Gimignano, Siena, Susa, Trizzino, Vagli, Vermiglio, Viareggio. From each record we extracted the following information: year, surnames, birthplace and, whenever possible, surnames and birthplaces of both parents. ISTAT original data were provided by the municipal offices of Montefegatesi (1993 records), Roggio (1993 records), Cannavo’ (2001 records) and Trizzino (2001 records). Original data from 1993 phone directories were kindly provided by SEAT in the form of magnetic tapes. Commercial data were subsequently filtered out. Each of present-day areas of jurisdiction (SEAT phone districts, ISTAT municipalities), largely overlap with the geographic extension pertained to the corresponding sacramental archive and comprise no more than one founding parish. In no case either population replacements or massive migrations occurred since the time of the historical source.

### Data analysis

An accurate context-specific transliteration of historical characters was done from original sources. Subsequently, a lemmatization of both “founding” and recent surnames was carried out. Namely, ancillary historical sources were used to correct for editing mistakes (i.e.”Bevlacqua = Bevilacqua”), single-digit mismatches (i.e. “Marino/Marini”) and double surnames (i.e. “Albertini Marchesani = Albertini”). In presence of doubtful cases, a stringent criterium was adopted in order to minimize the bias due to mutational events (i.e. “Benvenuti = Benvenutini”, “Rosso/Rossi”). Overall, such a method tends to inflate the degree of overlapping between “founding” and present-day distributions and, hence, to underestimate the sampling bias. The lemmatization process was applied separately to each community. The complete database is provided in S1 Dataset and counts 67,071 surnames and 310,571 individuals.

In order to validate the use of different sources, as either historical or recent references, we recovered and compared the surname distributions from SEAT and ISTAT records in the community of Cerchio (Abruzzo, Central Italy), as well the surname distributions from marriage and baptismal acts in the community of Montecarlo (Tuscany, Central Italy).

### Simulating sampling strategies

For each strategy we measured the sampling-dependent bias (SDB) according to the formula:

$$SDB=1-\frac{\sum_{k}^{1}x}{N_{k}},$$

where *x* is the number of the *k* surnames which are in common between the historical and current repository and *N*_*k*_ is the size of the current repository. SDB ranges from 1, no surname shared, to 0, all the surnames of the current repository are shared with the historical repository. A “low-kinship” (LK) model, namely when only one individual per surname was considered, and a “high-kinship” model (HK), when using the entire set of surnames and their bearers, were applied to each pairwise comparison. Under HK, *k* was intended as the total number of surnames, allowing multiple counts for the same type; under LK, *k* was intended as the number of different surnames, one for type. Being our null hypothesis the adhesion of the sampled surnames to the oldest surname distribution of each community, as occurs when collecting according to the founding surnames method, the SDB rate expectation is zero under both models.

To estimate how far the departures of SDB from zero are exclusively due to genetic drift we simulated the evolution of the 26 studied communities under both a growth (observed relative growth rates) and a stationary model (null growth rate) by the Markov chain Monte Carlo method implemented in the software ASHEs [16, 17]. For each model, 100 iterations were performed and averaged SDB values were compared with the observed values.

To estimate how far SDB is affected by sample size, we performed a total of 201 jackknife re-samplings of twenty, fifty and, when possible, one hundred individuals out of every dataset, using PopTools version 3.2.5 [18].

STATISTICA v. 6.0 (Stat-Soft Inc, Tulsa, OK) and XLSTAT (Addinsoft) were utilized to perform the Chi-square tests, the correlation analyses and to create graphics.

When simulating by the random sampling criterion (R), the entire list of surnames of the recent repository (SEAT or ISTAT) was considered as starting set. When selecting samples by a spatial distribution criterion (“locally spread” surnames or LS), we chose only those surnames whose geographic origin was unambiguously centered within a single province (the metropolises Milan and Rome excluded), as shown in the SEAT 1993-derived GENS database [19]. The surnames fitting such a criterion showed to be often, but not always, the rarest, with a number of bearers usually lower than 5,000. It is important to stress that local surnames satisfying this condition are not necessarily monophyletic, that is derived from a single ancestor. The frequency-based method (FQ) was applied by selecting the most frequent surnames, those falling above the upper quartile of the present-day distribution. The availability of marriage acts between 1845 and 1915, along with important information such as the place and date of birth of both the spouses and their parents, gave us the opportunity to select individuals by the grandparents’ criterion (GP), since this time interval represents the period when the grandparents of the 1991–1993 population lived. Simulating the “founding” surnames method (FS) meant to refer to the most ancient distribution found in the historical records. We actually used this sampling strategy as a template with SDB = 0 to compare the efficiency of other methods.

## Results

### Source-dependent bias

Bivariate analyses of present-day surname lists obtained from ISTAT (2001) and SEAT (1993) records in the community of Cerchio (S1A Fig) and of “founder” surname lists obtained from baptismal (A.D. 1527) and marriage (A.D. 1576) acts in the community of Montecarlo (S1B Fig) were performed. The two distributions, albeit differing as total number of surnames (SEAT = 168, ISTAT = 150; baptismal acts = 242, marriage acts = 224) and individuals (SEAT = 557, ISTAT = 481; baptismal acts = 2,310, marriage acts = 2,164), strongly correlate with each other (R^2^ = 0.87, P<0.01 and R^2^ = 0.82, P<0.01 respectively) and did not differ significantly (Chi-square test, P = 0.71 and P = 0.66 respectively). The fact that Cerchio and Montecarlo are communities with intermediate size and growth rate would suggest that, for most of the investigated communities, the availability of data sources exerted a negligible effect on the estimate of the intrinsic bias (SDB).

### Strategy-dependent bias

**Fig 2:**
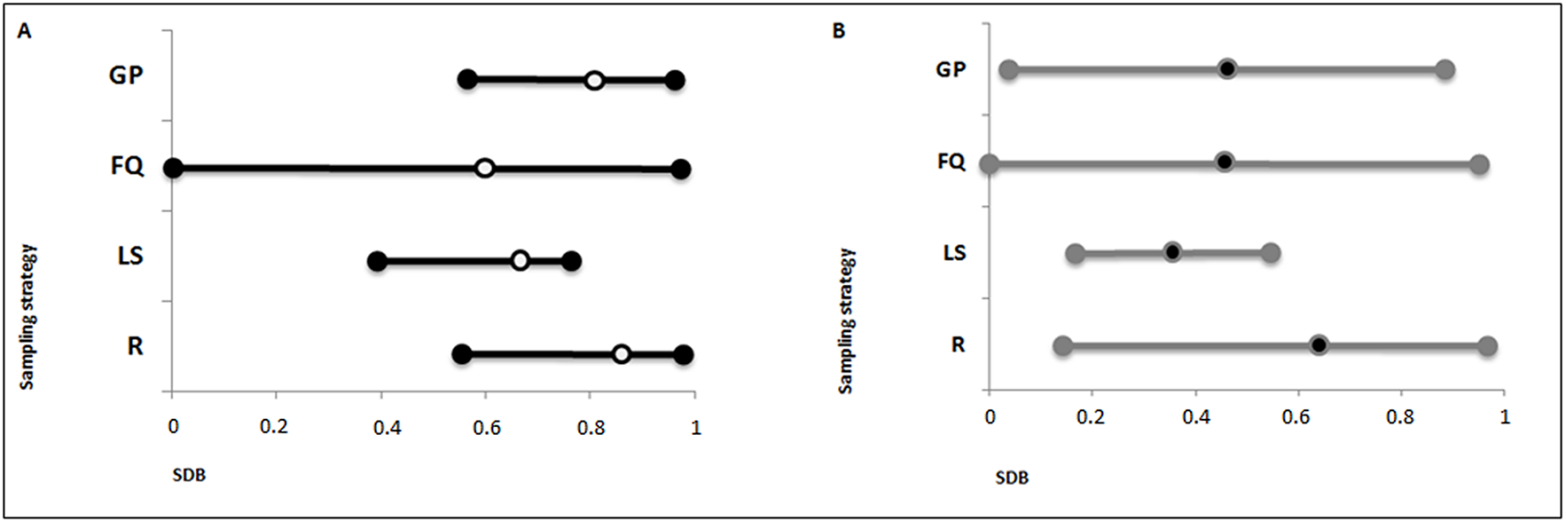
Minimum, maximum and mean values of the sampling-dependent bias (SDB) calculated after sampling by random (R), locally spread (LS), first quartile (FQ) and grandparents (GP) strategies, under the low-kinship (A) and the high-kinship (B) models in the 26 investigated communities.

As a rule, the comparisons between “founder” and present-day lists of surnames showed a low level of concordance independently of the applied sampling strategy and the kinship model (Fig 2A and 2B). Regarding the LK model, the observed average SDB was never below 0.595 (FQ strategy) with peaks of 0.770 and 0.846 (GP and R strategies, respectively). When multiple individuals per surname were considered (HK model), the average bias always decreased (by 8–30%) but at the cost of a larger variance.

The percentage of shared surnames sampled with a random strategy (R) was predictably low under a LK model (from 1 to 48%, SDB = 0.52–0.99) as well as under a HK model (from 2 to 83%, SDB = 0.17–0.98). Instead, it was somewhat surprising to observe the same features when applying the grandparents’ criterion (GP): surnames sharing ranged from 3 to 47% (SDB = 0.53–0.97) under the LK and from 6 to 95% (SDB = 0.05–0.94) under the HK.

The frequency-based criterion (FQ) showed the highest degree of uncertainty: surnames sharing, in fact, ranged from 4 to 100% (SDB = 0.00–0.96) under both the LK and the HK. The criterion based on locally-spread surnames (LS), on the other hand, showed the lowest bias: the overlapping degree varying from 22 to 65% under the LK (SDB = 0.35–0.78) and from 41 to 86% under the HK (SDB = 0.14–0.59). In this case preserved patrilineages approach the estimated proportion of “autochthony” of Italian surnames at the province level (from 22.8% to 77.9%, [5]). The selection of locally spread or rarer surnames, therefore, ensures a more similar albeit overall low degree of representativeness across communities. The historical residence of individuals bearing rare and localized family names, in fact, is not ensured because a focused spatial distribution may be the consequence of founder effects due to family renaming and/or migrations in the last few centuries.

**Fig 3:**
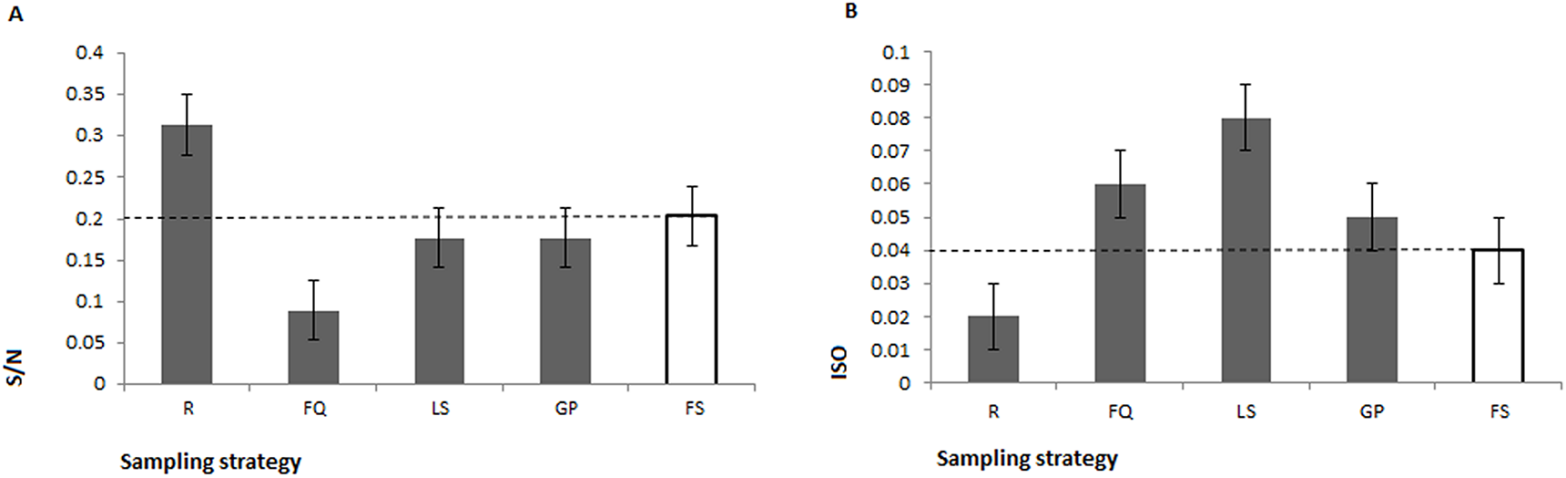
Average values and standard deviations of the S/N parameter (A) and isonymy (B) calculated with different sampling strategies in the present-day communities. S/N, relative number of surnames; ISO, isonymy. Sampling strategies: R, random; FQ, first quartile; LS, locally spread; GP, grandparents; FS, founder surnames.

In order to quantify how much the estimation of single population parameters is affected by the choice of the sampling method, we measured both the isonymy (according to [20]) and the value S/N, the relative number of different surnames S in present-day lists of surnames of size N, applying each strategy of sample selection in all the 26 communities. Such values differed with respect to the reference value calculated upon the FS method (sampling only “founding” surnames) depending on the strategy adopted (Fig 3A and 3B). Interestingly, the deviations observed were of inverse sign for the R and the other strategies, respectively inflating and deflating the ancestral diversity.

### Drift-dependent bias

Simulations performed under growth and stationary models allowed us to measure the expected bias accumulated by lineage sorting alone (genetic drift), from foundation to present times. As shown in S2 Fig, SDB rates observed by sampling randomly one individual per surname (R criterion under a LK model) gave a good estimation of the drift-dependent bias only in those small communities which underwent negligible migration (i.e. Careggine). The data in our possession (not shown) suggest that positive departures from this value are proportional to the cumulative impact of outwards and inwards migratory flows in all the other investigated communities.

### Community-dependent bias

A more accurate analysis was done by evaluating the dependence of SDB by the type of the community (S3–S6 Figs). A first observation is that the lowest SDB values were obtained in the alpine valleys of Trentino (North Italy). Here, the four communities investigated are known to show high levels of both consanguinity and emigration rates [21, 22]. For these communities, geographic factors likely reinforced isolation from the neighboring demes more than in other geographic contexts, thus facilitating the retention of surname-based legacies.

A second observation is the generally lower SDB values scored using HK compared to LK sampling criteria, with a more pronounced trend in small communities. The drop of SDB was significantly lower in communities with present-day size (N_t_) > 500 (Chi-square test, P<0.05). Similarly, we found that the higher the difference between the intrinsic bias calculated under the HK and LK methods (ΔSDB), the lower the value of S/N in the “founding” list, here used as a crude estimate of early population diversity [23]. This finding is somewhat expected because, in a stable population, the number of individuals is large when compared to the number of surnames and the effect is accentuated when the size is small because of drift. However, as shown in S7 Fig, a functional relation with S/N was found for the R (R^2^ = 0.35, P = 0.02), the FQ (R^2^ = 0.36, P = 0.02) and the GP (R^2^ = 0.78, P<0.001) sampling strategies but not for the LS criterion (R^2^ = 0.005, P = 0.96). The same tendency was observed between ΔSDB and isonymy, here intended as the random component of inbreeding (R^2^>0.35, P<0.05) (data not shown). Interestingly, Nonantola (Po river plain, North Italy), despite being not geographically isolated and with a size in the order of thousands, showed the highest ΔSDB when sampling by the FQ criterion. In this case, the retention of frequency-based surname legacies is more easily explained by its hosting a “Partecipanza”, a social-economic entity where rights over common lands have been long transmitted strictly following residency and patrilineal genealogies [24].

Lastly, we tested whether there could be any geographic or demographic trait of the community that would allow us to predict the sampling bias. We created a scatter plot showing the relationships between the intrinsic bias and altitude, present-day population size, growth rate and the time since the oldest repertoire of surnames (S8 Fig). Of the four variables tested, only altitude showed significant lines of tendency, even after multiple testing correction, under both low and high kinship models and for all the sampling strategies with the exception of the LS criterion (Table 2).

**Table 2:** Detailed list of R2 values and correlation signs (S) with nominal and Bonferroni adjusted P-values for different sampling strategies and models. Sampling strategies: R, random; FQ, first quartile; LS, locally spread; GP, grandparents; FS, founder surnames. Models: LK, low-kinship; HK, high-kinship.

Sampling strategy	Model	Covariate	R2	S	P-value	Adjusted P-value
R	LK	Altitude	0.42	-	0.00	0.008
	HK	Altitude	0.58	-	0.01	0.036
LS	LK	Altitude	0.01	-	0.98	1.000
	HK	Altitude	0.12	-	0.74	1.000
GP	LK	Altitude	0.36	-	0.01	0.036
	HK	Altitude	0.41	-	0.00	0.012
FQ	LK	Altitude	0.42	-	0.00	0.012
	HK	Altitude	0.43	-	0.00	0.008
R	LK	Present-day N	0.13	+	0.41	1.000
	HK	Present-day N	0.16	+	0.28	1.000
LS	LK	Present-day N	0.01	+	0.96	1.000
	HK	Present-day N	0.01	+	0.96	1.000
GP	LK	Present-day N	0.21	+	0.16	0.640
	HK	Present-day N	0.23	+	0.11	0.440
FQ	LK	Present-day N	0.11	+	0.46	1.000
	HK	Present-day N	0.12	+	0.42	1.000
R	LK	Foundation year	0.11	-	0.46	1.000
	HK	Foundation year	0.12	-	0.42	1.000
LS	LK	Foundation year	0.01	-	0.99	1.000
	HK	Foundation year	0.01	-	0.99	1.000
GP	LK	Foundation year	0.11	-	0.46	1.000
	HK	Foundation year	0.11	-	0.46	1.000
FQ	LK	Foundation year	0.02	-	0.89	1.000
	HK	Foundation year	0.02	-	0.89	1.000
R	LK	Growth rate	0.03	+	0.84	1.000
	HK	Growth rate	0.11	+	0.46	1.000
LS	LK	Growth rate	0.01	+	0.99	1.000
	HK	Growth rate	0.01	+	0.99	1.000
GP	LK	Growth rate	0.01	+	0.99	1.000
	HK	Growth rate	0.12	+	0.42	1.000
FQ	LK	Growth rate	0.01	+	0.99	1.000
	HK	Growth rate	0.09	+	0.54	1.000

### Scale-dependent bias

When the sampling bias was calculated at the level of mid (region) and macro (Country) area, the overall patterns observed at the municipality level held for the R and FQ sampling methods under both the LK and HK models (data not shown). Deviations are mainly due to the proportion of closed *vs* open communities in each region, which underestimates SDB in regions overrepresented by mountain villages (Trentino) and, *vice versa*, overestimates SDB in regions represented by large open cities (Apulia). Missing or overabundance of data prevented us to extend the analysis to higher levels than municipality, respectively for the GP and LS method. Nonetheless, a breakdown of SDB is expected when locally spread surnames are assigned at the regional and, of course, at the Country level.

### Size-dependent bias

The mean values of the percentage of “founder” surnames (1-SDB), calculated after randomly re-sampling twenty, fifty and one hundred individuals out of the complete list of present-day surnames, were not appreciably different under the LK (Chi-square test, P = 0.87) and HK (Chi-square test, P = 0.88) models. However, the range of values decreased geometrically by increasing the sample size.

The outputs of random Jackknife resampling for the S/N parameter are shown in S9 Fig, taking Bagaladi (South Italy) as an example of the general tendency in the other twenty six communities (LK, Chi-square test, P = 0.91; HK, Chi-square test, P = 0.93). This tendency shows that only samples approaching N = 100 ensure a reliable estimate of diversity.

## Discussion

The identification of the most reliable sampling strategy, among the many available for human genetic studies, could be of great utility. Conversely, an improper collection of samples from the population may introduce hidden confounding factors. With this perspective, we calculated the various sources of bias associated with common methods of sample recruitment: those employing family names as ancestry markers. Sampling by surnames is in fact one of the most accurate and cost-efficient ways to assess the genetic composition of historical populations, despite the fact that family names behave as a single locus transmitted along only one parental lineage, and the fact that mismatches are expected in Y-DNAs due to illegitimate paternities and adoptions [25].

A main result of our analyses is that the fidelity to the ancestral composition of the population (measured from 1447–1775) by samples selected from current lists of surnames is variable but generally low whatever is the size of the sample, the selection strategy, the primary source of data, the geographic scale, or type of the community. As expected, lineage sorting accounts for the deviations from fidelity only partially and in very closed communities.

Despite the use of a relaxed lemmatization, biases lower than 40%, intending this term as the complement to one of the degree of overlapping between “founding” and present-day lists of family names (SDB), have been observed only in small mountainous hamlets as far as a close parenthood among participants was admitted (high-kinship model). The selection of unrelated donors is usually associated with a substantial increase of the bias other than to higher costs of recruitment and, in remote areas, a lower sample size. Low kinship in most cases means rates of bias much higher than 50%, unless the first quartile method is applied in stable communities where cultural (socio-economic rules, language) or geographic (mountain terrain) barriers preserved family names with high frequency over long periods of time.

Population stratification due to recent fluctuations in allele frequencies over time thus appears a foremost level of apportionment of the overall genetic variance. While typically not recognized because of the lack of contribution of bio-demographic data to genetic sampling designs, the diachronic variance would play a crucial role in the representativeness of a population sample and, hence, to its utility in biological research.

The proportion of ancestry from ancient and recent demography can vary widely between individuals, leading to step-ups of the internal structure of the population which, in turn, may easily introduce either spurious results (type I and II errors) in genetic association studies or distortions in historical and evolutionary reconstructions. The latter case has been demonstrated by Manni et al. [4] who found that, sampling Dutch Y chromosomes without a prior selection of donors on the base of surnames’ origin, led to skewed population structures. In addition, Bowden et al. [26] demonstrated that independent samples ascertained on the basis of residency (GP method) and on the possession of medieval surnames (FS) showed significantly different Y-haplotypes. Similarly, Calò et al. [27] showed how different sampling strategies, founding surnames (FS) and the grandparents’ (GP) methods, can lead to contrasting population affinities on the basis of mitochondrial HVRI haplotypes.

Here, we demonstrated that sampling criteria could easily affect population parameters resulting in inflated (random methods) or deflated (selective methods) estimates of diversity.

The most efficient method to correct population parameters for the diachronic bias is to select individuals bearing historically documented surnames (“founders” method). Alternative but equally time-demanding methods, such as those based on surname distribution (LS) and residency (GP), are heavily influenced by recent gene flows and appeared to be efficient only in specific contexts, as macro geographic scales and alpine villages. Moreover, they generally result in a reduction of the maximum gatherable sample size down to a hundred or even less. Consequently, their use in population genetics is limited by the poor statistical power, and is very limited or impracticable in genotype-phenotype association studies. In these latter cases a truly random sampling under a HK model, despite it carrying a high SDB, is able to give samples of suitable size at affordable costs.

Among the various features used to define a human community both geographically and demographically, altitude was found the best proxy of the bias inherent to most of the sampling procedures in the modern Italian population.

We can conclude that sampling individuals bearing historically documented surnames should be the method of choice to buffer the stratification of ancient and recent genetic components of the sampled population. Nonetheless, the selection of “founders”, while excluding misleading results from markers of the male specific fraction of the Y chromosome, does not prevent that recent population reshufflings heavily driven by women mobility could bias inferences and statistics based on the variability at autosomal, X-chromosome and mitochondrial markers.

## Supporting Information

S1 Fig Number of individuals (N) per surname, comparing data sources from ISTAT and SEAT records (Fig A) and from baptismal and marriage acts records (Fig B). (TIF)

S2 Fig Observed (red dots) and simulated values of SDB sampling randomly under a low-kinship model in the 26 investigated communities. Yellow lines, simulated SDB values with population growth; yellow diamonds, simulated SDB values with constant population size.
(TIF)

S3 Fig Sampling-dependent bias (SDB) calculated after sampling by the random strategy in each of the 26 investigated communities. HK, high-kinship model; LK, low-kinship model.
(TIF)

S4 Fig Sampling-dependent bias (SDB) calculated after sampling by the first quartile strategy. HK, high-kinship model; LK, low-kinship model.
(TIF)

S5 Fig Sampling-dependent bias (SDB) calculated after sampling by the grandparents strategy. HK, high-kinship model; LK, low-kinship model.
(TIF)

S6 Fig Sampling-dependent bias (SDB) calculated after sampling by the locally spread strategy in the investigated communities. HK, high-kinship model; LK, low-kinship model.
(TIF)

S7 Fig Correlation between ΔSDB and S/N values under the random (A), first quartile (B), locally spread (C) and grandparents (D) methods. (TIF)

S8 Fig Plots showing correlation between communities’ features (altitude, present-day population size, year of the oldest list of surnames, growth rate) and sampling-dependent bias (SDB), calculated sampling by random (R), locally spread (LS), first quartile (FQ), grandparents (GP) strategies under low-kinship (LK) (A) and high-kinship (HK) (B) models. (TIF)

S9 Fig Variation of the S/N parameter with different Jackknife re-sampled population sizes in the present-day population of Bagaladi, following the high-kinship (A) and low-kinship (B) models. (TIF)

S1 Dataset Complete surnames database. (XLSX)
