# Supplementary figures and images for: Estimating Sampling Selection Bias in Human Genetics: A Phenomenological Approach
Source: PLoS One. 2015 Oct 9;10(10):e0140146. doi: 10.1371/journal.pone.0140146 (PMC4599962; doi:10.1371/journal.pone.0140146)

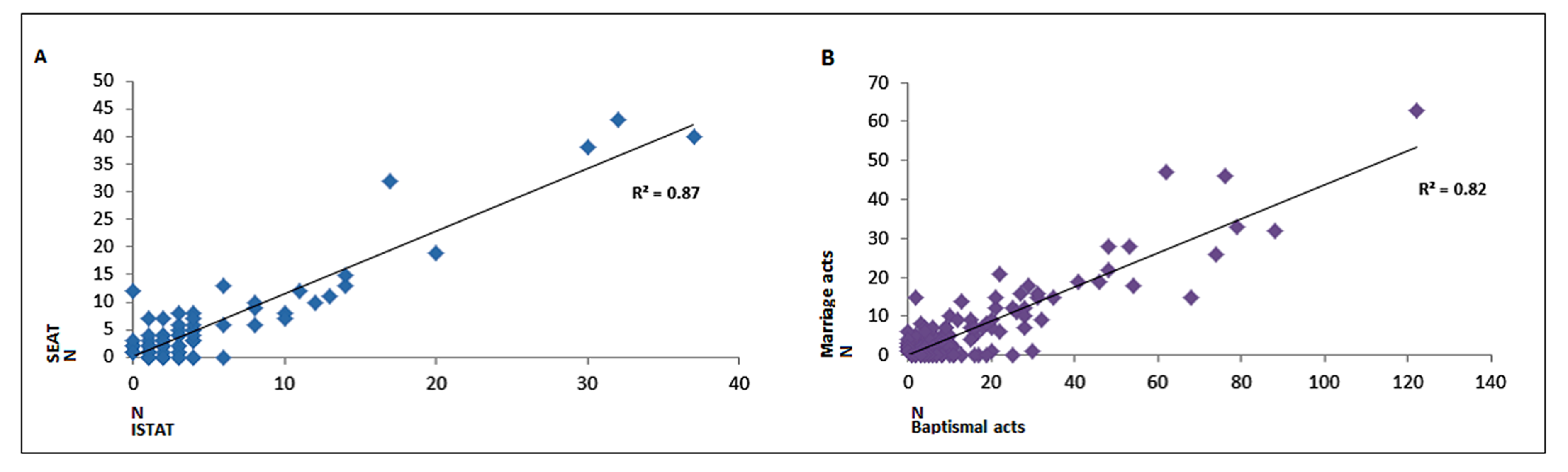

Supplement: S1 Fig — (TIF) [file pone.0140146.s001.tif]

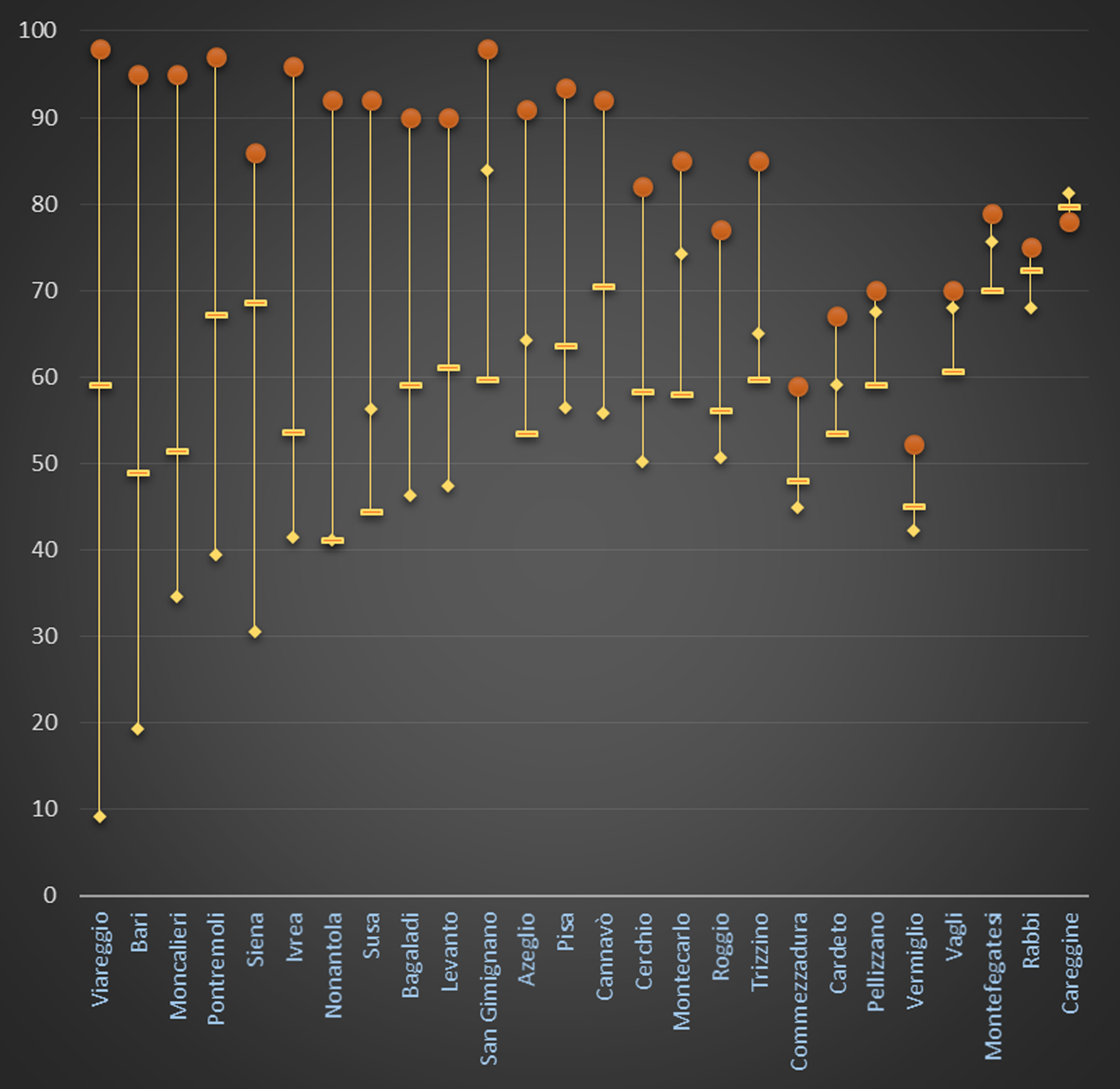

Supplement: S2 Fig — Yellow lines, simulated SDB values with population growth; yellow diamonds, simulated SDB values with constant population size. (TIF) [file pone.0140146.s002.tif]

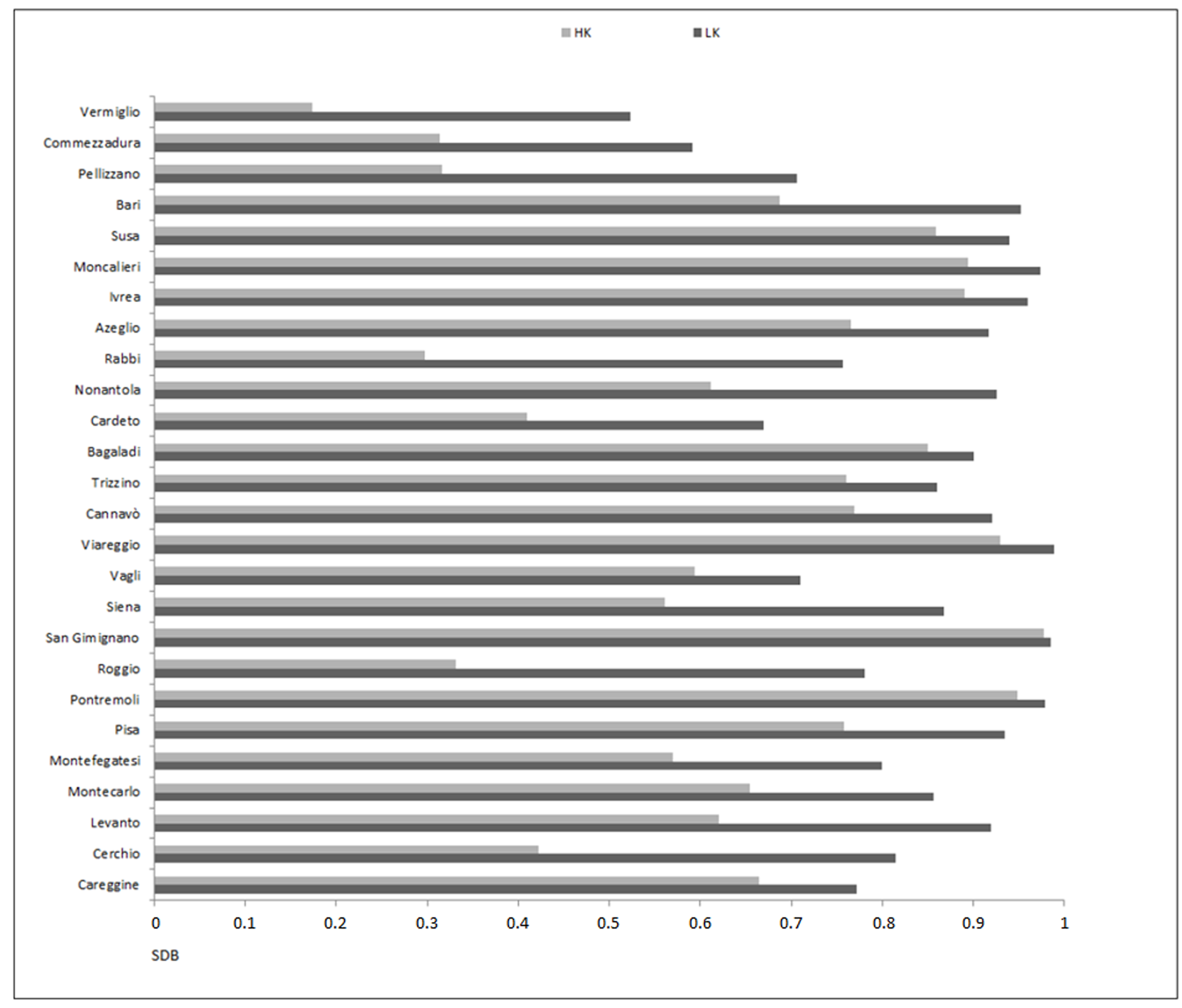

Supplement: S3 Fig — HK, high-kinship model; LK, low-kinship model. (TIF) [file pone.0140146.s003.tif]

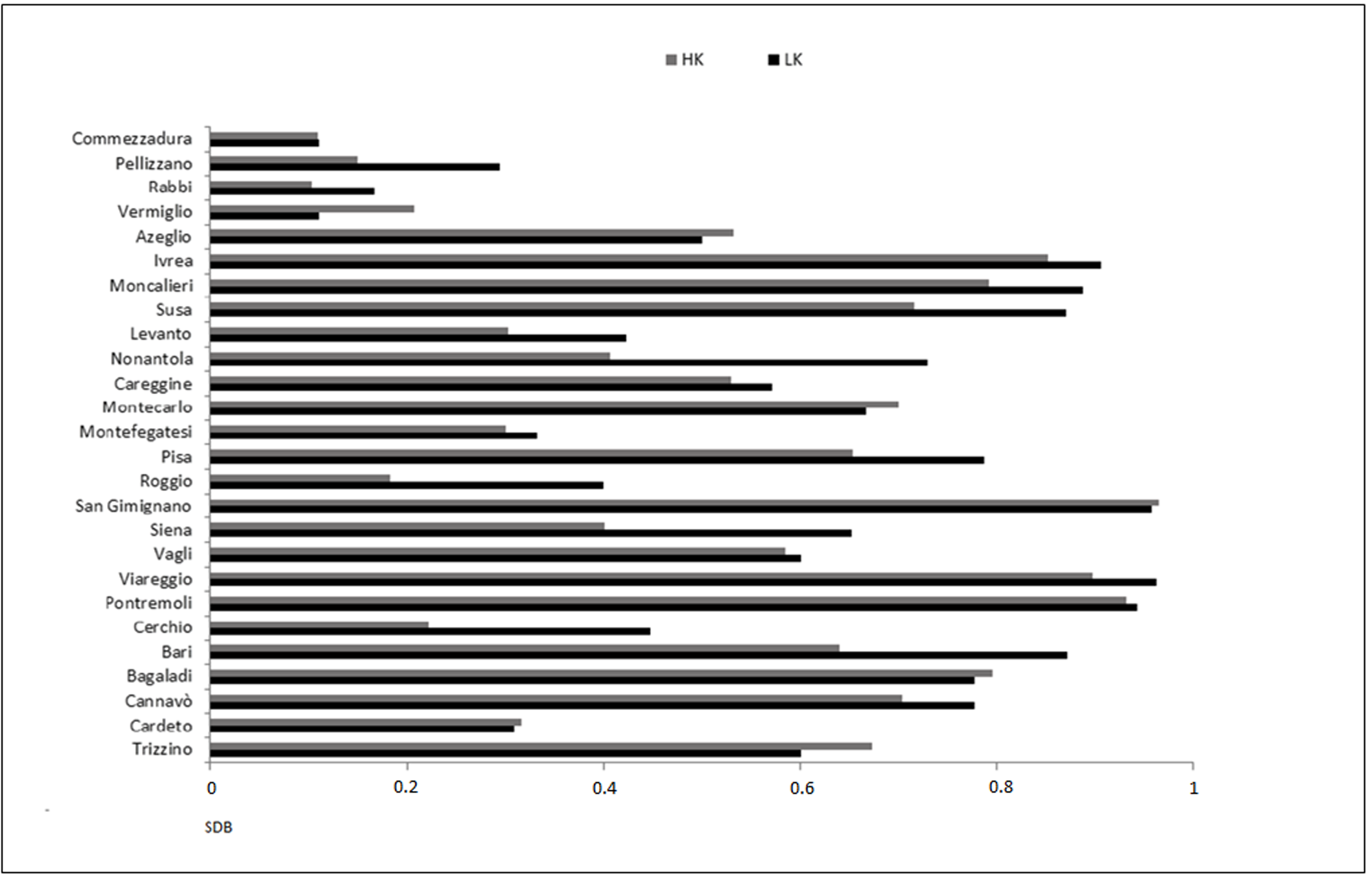

Supplement: S4 Fig — HK, high-kinship model; LK, low-kinship model. (TIF) [file pone.0140146.s004.tif]

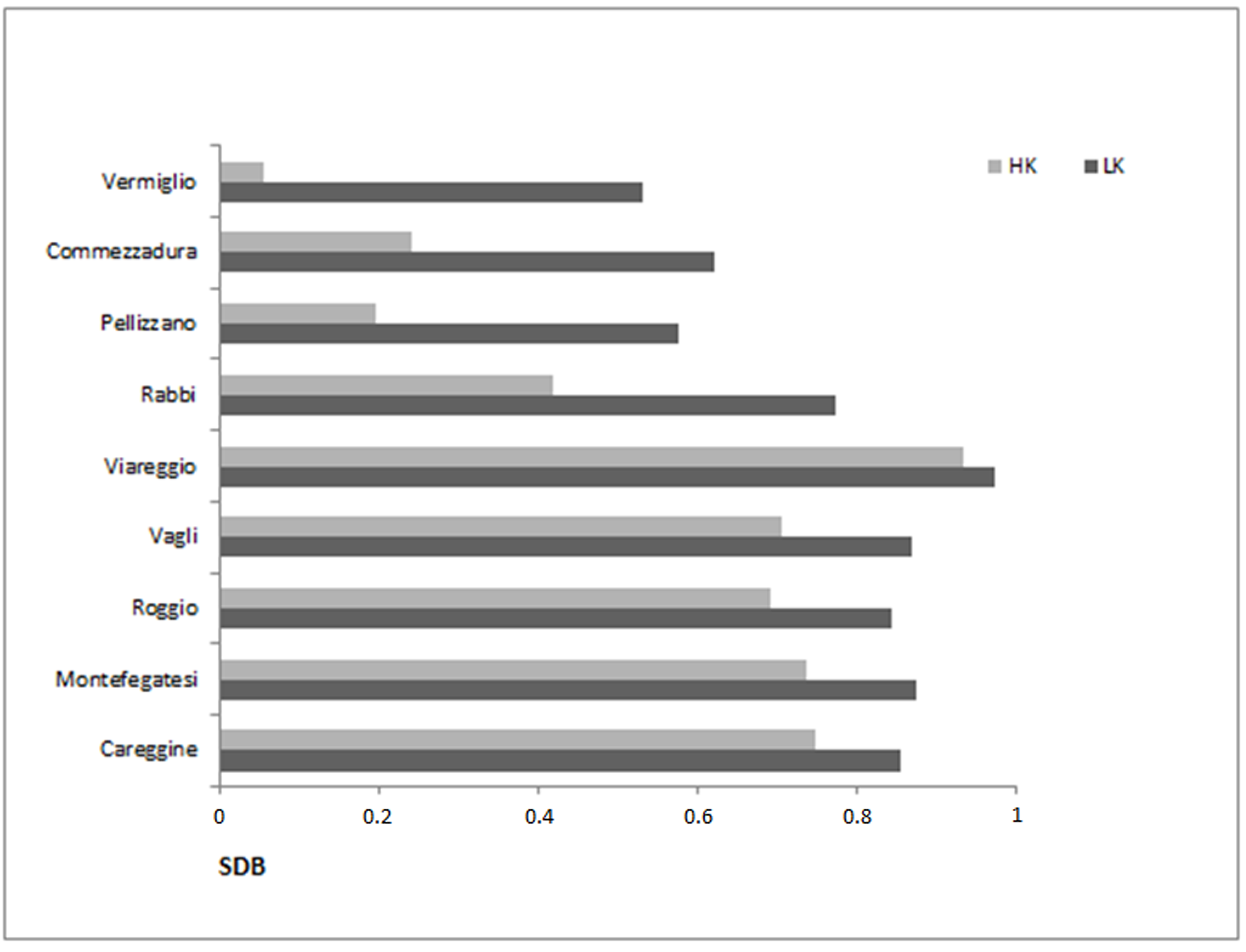

Supplement: S5 Fig — HK, high-kinship model; LK, low-kinship model. (TIF) [file pone.0140146.s005.tif]

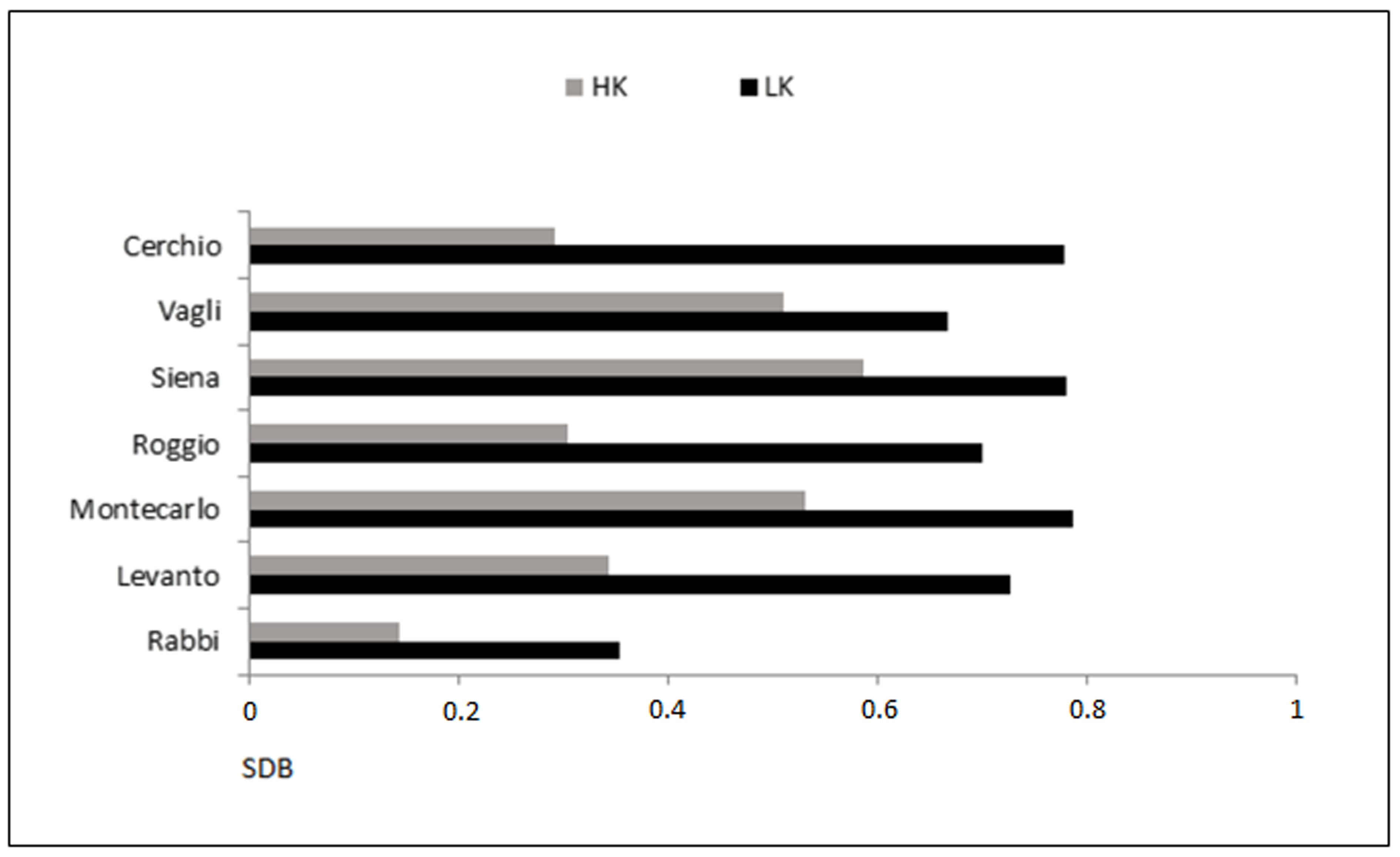

Supplement: S6 Fig — HK, high-kinship model; LK, low-kinship model. (TIF) [file pone.0140146.s006.tif]

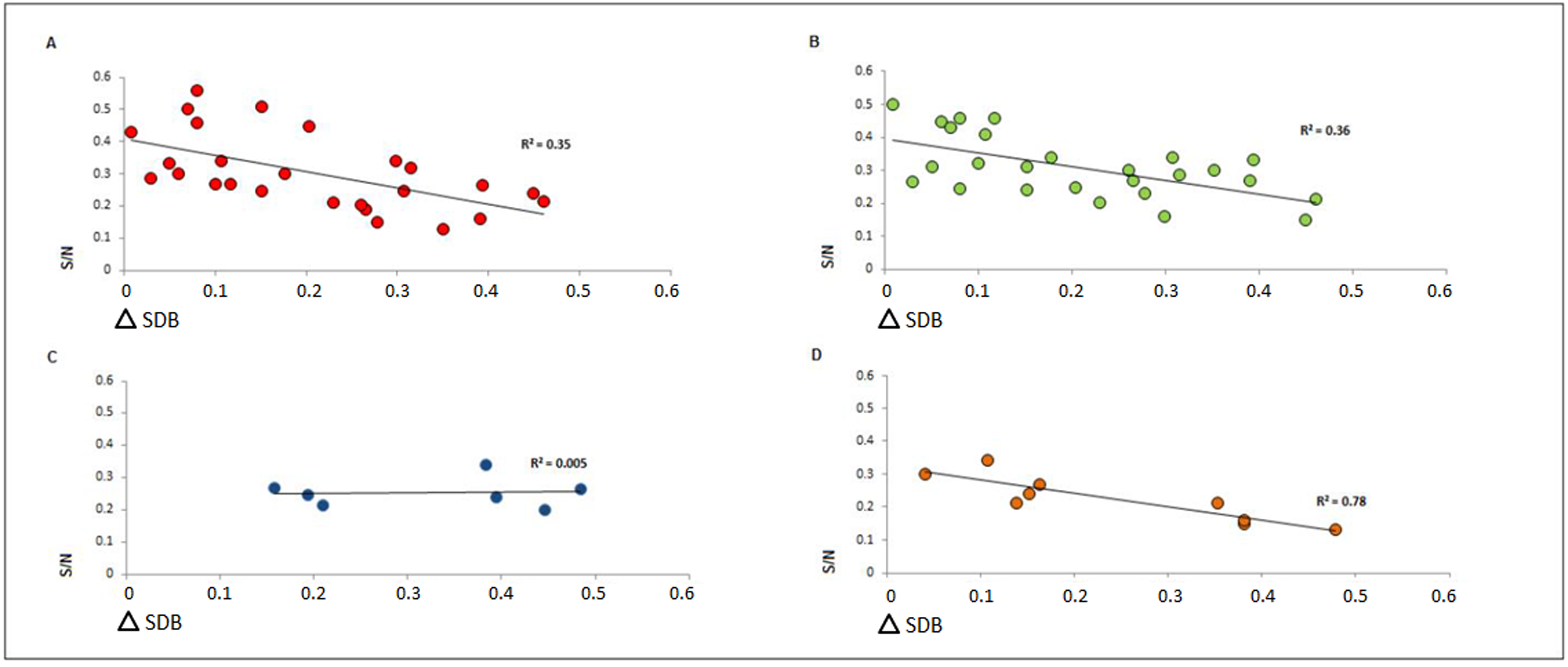

Supplement: S7 Fig — (TIF) [file pone.0140146.s007.tif]

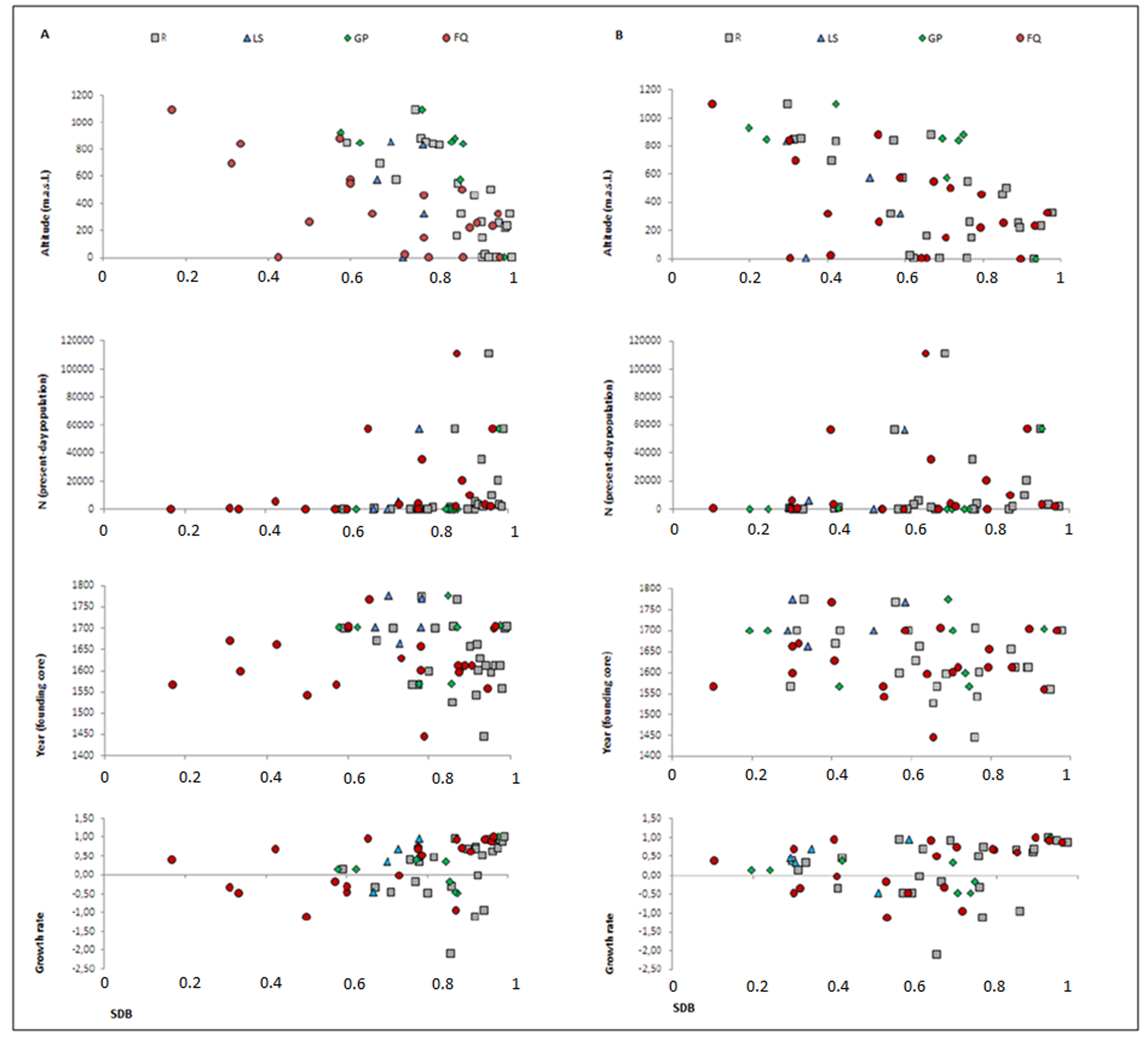

Supplement: S8 Fig — (TIF) [file pone.0140146.s008.tif]

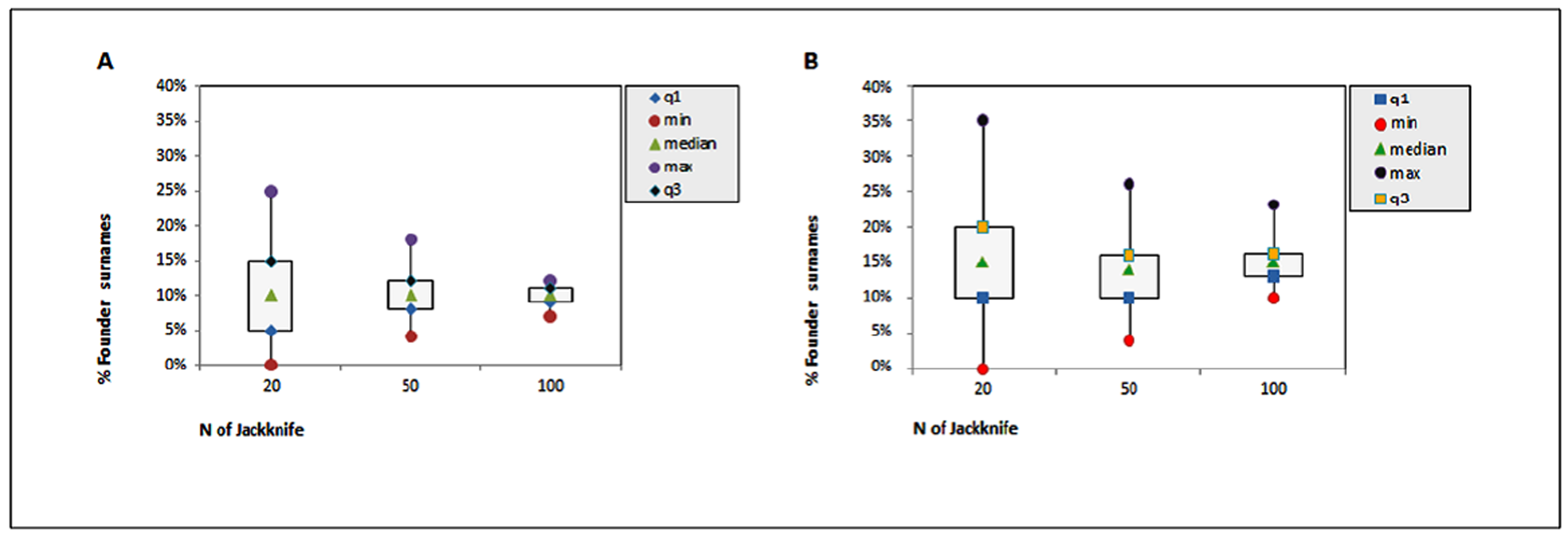

Supplement: S9 Fig — (TIF) [file pone.0140146.s009.tif]
